# Role of procalcitonin in diagnosis of community acquired pneumonia in Children

**DOI:** 10.1186/s12887-022-03286-2

**Published:** 2022-04-20

**Authors:** Vinod H. Ratageri, Puspha Panigatti, Aparna Mukherjee, Rashmi R. Das, Jagdish Prasad Goyal, Javeed Iqbal Bhat, Bhadresh Vyas, Rakesh Lodha, Deepak Singhal, Prawin Kumar, Kuldeep Singh, Samarendra Mahapatro, Bashir Ahmad Charoo, S. K. Kabra, K. R. Jat

**Affiliations:** 1 Department of Pediatrics, Karnataka Institute of Medical Sciences, Hubballi, 580021 Karnataka India; 2 Department of Pediatrics, All India Institute of Medical Sciences, New Delhi, India; 3 Department of Pediatrics, All India Institute of Medical Sciences, Bhubaneswar, India; 4 Department of Pediatrics, All India Institute of Medical Sciences, Jodhpur, India; 5 Department of Pediatrics, Sher I Kashmir Institute of Medical Sciences, Srinagar, Jammu and Kashmir, India; 6 Department of Pediatrics, MP Shah Medical College, Jamnagar, Gujrat India

**Keywords:** Biomarkers, Children, Pneumonia, Procalcitonin, Acute respiratory infection

## Abstract

**Background:**

The role of serum Procalcitonin (PCT) in adults in diagnosis of Community acquired pneumonia (CAP) is well established, however, role in pediatric CAP remains controversial.

**Objectives:**

The objective of this study was to investigate the utility of serum procalcitonin in differentiating bacterial community-acquired lower respiratory tract infection from non-bacterial respiratory infection in children; radiologically confirmed pneumonia was used as the reference. In addition, we assessed the utility of adding the PCT assay to the clinical criteria for diagnosis of pneumonia.

**Study design:**

Subanalysis of a larger prospective,multicentriccohort study.

**Participants:**

Children, 2 months to 59 months of age, attending paediatric OPD of 5 urban tertiary care hospitals, suffering from acute respiratory infection (ARI).

**Intervention:**

Detailed clinical history and examination findings of enrolled children were recorded on predesigned case record form. Samples for PCT were obtained at admission and were measured centrally at the end of the study except for one site using VIDAS® B.R.A.H.M.S PCT kit (Biomerieux SA, France).

**Outcomes:**

Sensitivity and specificity of procalcitonin for diagnosis of radiologically confirmed pneumonia.

**Results:**

Serum Procalcitonin was measured in 370 patients; median (IQR) age of these children being 12 (7, 22) months, 235 (63.5%) were boys. The median (IQR) serum procalcitonin concentration was 0.1(0.05, 0.4) ng/mL.Sensitivity and specificity of raised PCT (> 0.5 ng/mL) for pneumonia as per any CXR abnormalities were 29.7% and87.5%,(*P* < 0.001) respectively. Raised PCT was also significantly associated with consolidation (34.5%,79.2%,*P* < 0.02)and pleural effusion(54.6%,79%,*P* < 001)**.** Adding PCT to the existing clinical criteria of WHO did not improve the sensitivity for diagnosis of pneumonia. PCT was significantly higher in children with severe pneumonia.

**Conclusion:**

Positive PCT (> 0.5 ng/mL) is significantly associated with radiographic pneumonia but not with pneumonia based on WHO criteria.However, it can act as a surrogate marker for severe pneumonia.

## Introduction

Community-acquired pneumonia (CAP) is a common clinical problem in childhood [1]. Bacterial pneumonia cannot be differentiated from viral pneumonia based on clinical or chest radiographic findings [2]. The use of biomarkers in clinical practice has increased substantially specially because proponents claim that biomarkers may improve the early diagnosis of infections and be available as a point-of-care tool [3]. This may allow earlier and better identification and treatment of patients with severe life-threatening infections.

Procalcitonin(PCT) is a protein, precursor of hormone Procalcitonin, normally produced by neuroedocrine cells present in thyroid, lung and intestine [4, 5]. Procalcitonin (PCT) has been introduced as a marker of bacterial infection, having been used to differentiate between septic and other infections in pediatric patients [6]. In many adult studies, it’s been well established that higher PCT concentration is associated with isolation of typical bacterial organisms [7, 8], especially, in relation to bacteraemic CAP [9, 10]. However,same evidence is lacking in children. Two studies in children [11, 12] found limited association between PCT concentration and identification of organisms in CAP where as in other studies found reasonably good association [13, 14]. Many previous studies were limited by sensitivity of PCT assays, identification of organisms and testing methods. The objective of this study was to investigate the utility of serum procalcitonin in differentiating bacterial community-acquired lower respiratory tract infection from non-bacterial respiratory infection in children; radiologically confirmed pneumonia was used as the reference. In addition, we assessed the utility of adding the PCT assay to the clinical criteria for diagnosis of pneumonia.

## Methodology

### Study design

Prospective cohort study. The currently reported study is a part of a larger study designed to evaluate the effectiveness of acute respiratory infection treatment unit (ATU) in management of acute respiratory tract infection (ARI).

### Settings

Study was carried out at the following five urban tertiary care hospitals of India: 1. Sher-I-Kashmir Institute of Medical Sciences (SKIMS), Srinagar 2. All India Institute of Medical sciences (AIIMS), Jodhpur, 3. AIIMS, Bhubaneshwar 4. Karnataka Institute of Medical Sciences, Hubbali, Karnataka 5. MP Saha Medical College, Jamnagar; AIIMS, New Delhi was the coordinating centre.

### Study duration

Two years (from June 2016 to May 2018).

The methodology of the main study has been described in previous publications [15–17]. Briefly, previously healthy children, between 2 to 59 months of age with ARI were enrolled in the main study. Any cough and/or breathing difficulty for duration of less than 2 weeks were considered as ARI. Procalcitonin estimation was done in subset of patients with clinical pneumonia based on WHO criteria [18] at the time of admission.

Chest radiograph was done in all cases of clinical pneumonia and every fifth child with no pneumonia due to limited budget at the time of enrolment plus avoidance of unnecessary radiation exposure in children with URI.

### Target condition and reference standard

Community acquired pneumonia (CAP) was target condition and radiological diagnosis was used as reference standard.

### Primary outcome

Children with abnormal chest radiograph was considered suggestive of bacterial pneumonia. Sensitivity, specificity and likelihood ratios were calculated for different levels of serum procalcitonin levels for diagnosis of pneumonia, using radiological confirmed pneumonia as the reference.

## Sample Size

As it was a sub-analysis, we did not perform sample size calculations for this separately. Within the budget availability, the sample size from all the sites for PCT was estimated to be 370 in children who were expected to be subjected to X ray chest.

## Diagnosis of pneumonia

### Clinical pneumonia

Clinical diagnosis of pneumonia was made by the physician before reading the CXR. The following WHO criteria were used for diagnosis of pneumonia (15):

1. cough or difficulty breathing and
2. age-specific tachypnea (≥ 50 breaths per minute for children 2–11 months of age and ≥ 40 breaths per minute for children 1–5 years of age).

### Severe pneumonia

Along with presence of age specific fast breathing and/ or chest in drawing, Children with the presence of any danger signs like lethargy, inability to drink, drowsiness, were classified as severe pneumonia.

### Radiologically confirmed pneumonia

It was defined as presence of pulmonary infiltrate or consolidation. Additional findings of interstitial infiltrates, peribronchial thickening and atelectasis were also recorded.

## Chest X-ray

As there were no consensus for reference standard for diagnosis of pneumonia, in this study, we have taken chest radiographic abnormality as gold standard.Chest radiograph (an anterior–posterior or posterior-anterior view), preferably digital (analogue if digital is not available) was done in all cases of clinical pneumonia. The radiographs was interpreted by site investigator at the time of enrolment and appropriate treatment to the patient was administered as per WHO guideline. The films of chest X ray were digitalisedor soft copy of the digital x-ray or hard copy of X rays were sent to the coordinating centre at the All India Institute of Medical Sciences, New Delhi. All CXR were read by two independent pediatricians(SKK/KRJ), who were blinded for the clinical diagnosis of patient. In case of disagreement about the presence or absence of pneumonia, chest x-rays were read by a third pediatrician(RL) without knowledge of the previous evaluations and final findings matching for two of them were considered final for purpose of analysis.

## Blood tests

It had been planned to collect two mL blood for procalcitonin (PCT) estimation at the time of admissionfrom all children with suspected pneumonia enrolled at all the sites, however due to budget constraints only 370 samples could be collected. The serum samples were kept frozen at -80 °C in respective centres before they were transported in a cold chain to AIIMS, New Delhi(from three sites AIIMS Bhubaneshwar; KIMS, Hubballi; and SKIMS, Srinagar) while samples collected at Jodhpur were tested locally.At AIIMS, Delhi, as well as in Jodhpur, the procalcitonin (PCT) assay was done using VIDAS® B.R.A.H.M.S. PCT kit (Biomerieux SA, France). Cut-off for positive serum procalcitonin level (PCT + ve) has been considered at > 0.5 ng/mL and PCT –ve if serum procalcitonin level ≤ 0.5 ng/mL. The cut-off was based on earlier studies [19, 20].

## Ethical considerations

Ethical approval was obtained from Ethics Committee of KIMS and other five study sites.

Written informed consent was obtained from legal guardians/parents of participants. A copy of consent form was given to parents/ guardian. Personal information (paper and electronic registers) were stored in an appropriate manner to ensure full confidentiality.

### Statistical analysis

All data were entered in Microsoft Access software and analysed by statistical software STATAv.14 (College Station, TX, US). Sensitivity, specificity, likelihood ratios (positive and negative) and predictive values (positive and negative) were calculated for PCT considering the radiographic pneumonia as the standard for diagnosis of pneumonia. Receiver operating characteristic (ROC) curve was used to evaluate the ability of PCT to differentiate between pneumonia and no pneumonia diagnosed by various criteria.

## Results

**Fig. 1:**
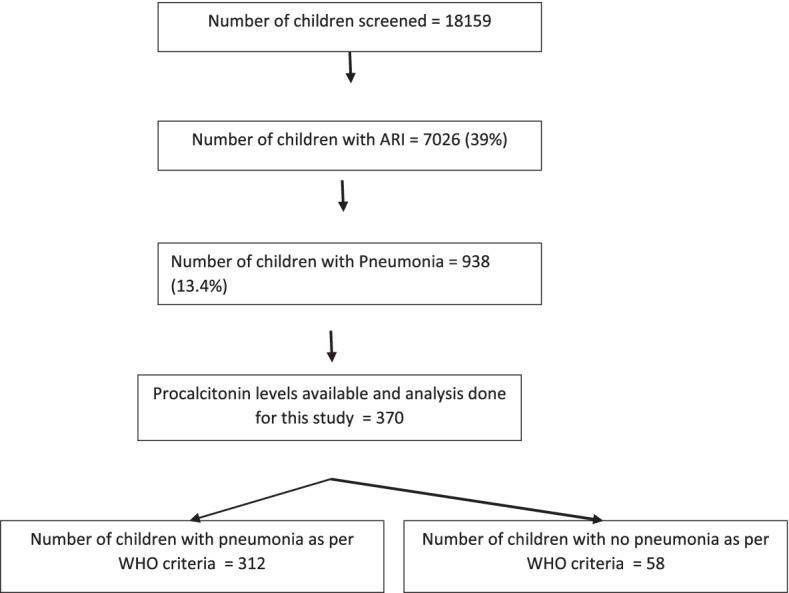
Flow chart of children screened and enrolled in the study

A total 7026 children with ARI (39% of screened children) were enrolled in the main study (Fig. 1). According to the WHO criteria, 13.4% (938) had pneumonia [37%(347) of them had severe pneumonia].Serum Procalcitonin was measured in 370 (235 boys) patients; median (IQR) age of these children being 12 (7, 22) months. Pneumonia as per WHO criteria was present in 312/370 children. The median (IQR) serum procalcitonin concentration was 0.1(0.05, 0.4) ng/mL.Positive PCT level (> 0.5 ng/mL) was seen in 83 (22.4%) children.

The demographic and clinical details of children in whom PCT level were measured are shown in Table 1. Of these children, 87.9% had pneumonia with raised PCT level as per WHO clinical case definition., However, 83.3% of those with normal PCT (< 0.5 ng/ml) also had pneumonia.

**Table 1:** Demographic and clinical details of children in whom PCT levels were measured, *n* = 370

**Parameter**	**Children with PCT > 0.5 ng/mL, *n* = 83**	**Children with PCT ≤ 0.5 ng/mL, *n* = 287**	**All enrolled children, *n* = 370**
Age in months, median (IQR)	12 (7, 19)	12 (6,24)	12 (7, 22)
Boys, n (%)	55 (66.3)	180 (62.7)	235 (63.5)
Weight for height/length z score, median (IQR)	-1.05 (-2.25, -0.09)	-1.11 (2.31, 0)	-1.07 (-2.31, 0)
WHO pneumonia n(%)			
Present	73 (87.9)	239 (83.3)	312 (84.3)
Absent	10 (12.1)	48 (16.7)	58 (15.7)

The association of PCT with chest X-ray abnormalities is detailed in Table 2. In the original study evaluating the effectiveness of ATU in treatment of ARI, 1273 CXR were readable. In the subset of 370 children, who had PCT levels estimated (inclusion criteria for this study), 337 CXRs were readable. Positive PCT is significantly associated with any abnormality on CXR (the gold standard of diagnosis of pneumonia). The sensitivity and specificity were 29.7% and 87.5% respectively (*P* < 0.001). And also consolidation on chest X-ray is significant(*P* < 0.02). However, PCT is not significantly associated with the existing WHO criteria for clinical pneumonia.

**Table 2:** Association between serum procalcitonin (PCT) concentrations and pneumonia as per CXR pathologyand existing WHO classification in 370 children in whom PCT levels are available

**Any abnormality on chest X-ray**			
	Abnormality present on CXR	No abnormality on CXR	Total
PCT + ve	62 **(29.7)**	16 (12.5)	78
PCT –ve	147 (70.3)	112 **(87.5)**	259
Total	209 (100)	128 (100)	*N* = 337
			*P* < 0.001
**Consolidation on chest X-ray**			
	Consolidation present	Consolidation absent	Total
PCT + ve	20 **(34.5)**	58 (20.8)	78
PCT –ve	38 (65.5)	221 **(79.2)**	259
Total	58 (100)	279 (100)	*N* = 337
			*P* = 0.02
**Pneumonia as per WHO criteria**			
	WHO pneumonia	No pneumonia	Total
PCT + ve	73 **(23.4)**	10 (17.2)	83
PCT –ve	239 (76.6)	48 **(82.8)**	287
Total	312 (100)	58 (100)	*N* = 370
			*P* = 0.30

PCT + ve: Children with serum PCT concentrations > 0.5 ng/mL;CXR: Chest X-ray, only 337 CXRs were available as not all children without pneumonia underwent CXR

All values are expressed as n (%)

**Fig. 2:**
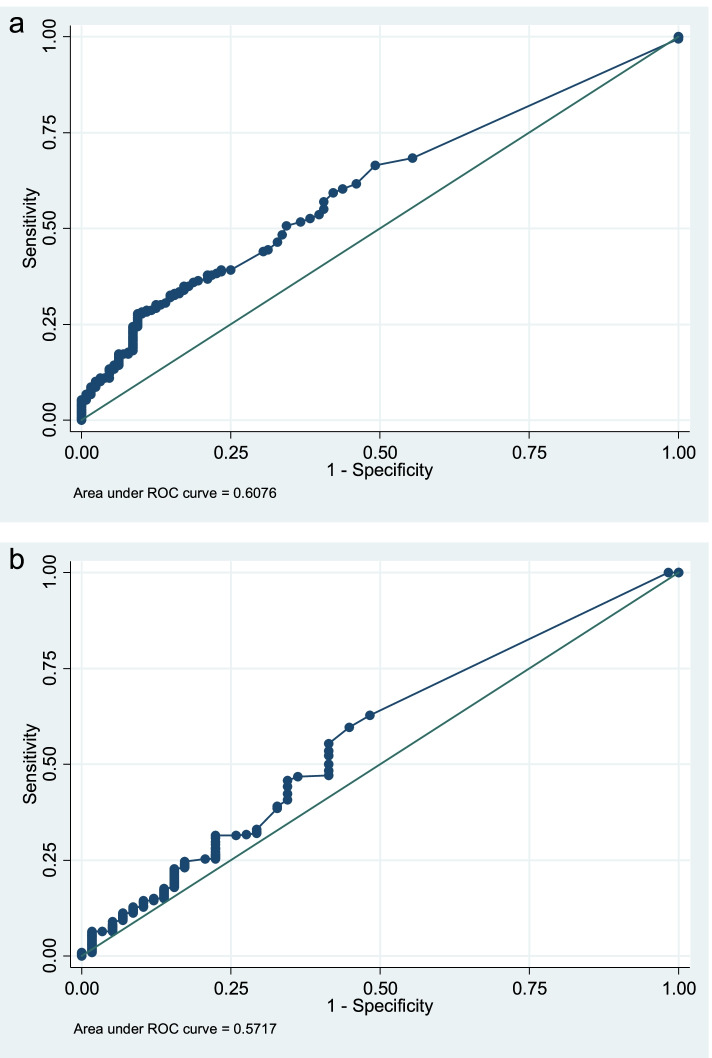
**a** PCT level with any abnormality on CXR as the classifier. **b** PCT level in ng/mL with WHO pneumonia as the classifier

Figure 2 (a, b) shows ROC for PCT in relation to any abnormality on CXR (AUC: 0.61) and WHO clinical Pneumonia (area under curve(AUC): 0.57.Sensitivity, specificity, predictive values, likelihood ratios of PCT for pneumonia diagnosed by different criteria are shown in Table 3.

**Table 3:** Sensitivity, Specificity, Predictive value, Likelihood ratio of Procalcitonin (> 0.5 ng/mL) for different diagnostic criteria of pneumonia

	Sensitivity	Specificity	Likelihood ratio positive	Likelihood ratio negative	Positive predictive value	Negative predictive value
Pneumonia as per any CXR abnormalities	29.7 (23.6, 36.4)	87.5 (80.5, 92.7)	2.4 (1.4, 3.9)	0.8 (0.7, 0.9)	79.5 (70.1, 86.5)	43.2 (40.6, 45.9)
Pneumonia as per presence of consolidation on CXR	34.5 (22.5, 48.1)	79.2 (73.9, 83.8)	1.7 (1.1, 2.5)	0.8 (0.7, 1.01)	25.6 (18.4, 34.5)	85.3 (82.7, 87.6)
Pneumonia as per WHO criteria	23.4(18.8, 28.5)	82.8 (70.6, 91.4)	1.4 (0.7, 2.5, 7.8)	0.9 (0.8, 1.1)	87.9 (80.5, 93)	16.7 (14.9, 18.6)

PCT + ve: Cut-off for positive serum procalcitonin level has been considered at > 0.5 ng/mL, (Values are in % with 95% CI) except likelihood ratios

PCT levels were significantly higher in the 22 children with uncomplicated para-pneumonic pleural effusion [0.58 (0.17, 4.02) ng/mL vs. 0.1 (0.05, 0.35); *p* = 0.001] as compared to those who had no pleural effusion.

Tables 4 and 5 shows comparison of PCT (at different cut-offs) between any abnormality in CXR and no abnormality and non severe pneumonia and severe pneumonia (as per WHO clinical criteria, respectively. The PCT (> 0.25 ng/ml) is significantly associated with any abnormality in CXR, however no significant difference observed between severe and non severe pneumonia.

**Table 4:** Comparison of serum procalcitonin level (at different cut-offs) between any abnormality in CXR and no abnormality

	Any abnormality on CXR	No CXR abnormality	*p*
PCT level > 0.25 ng/ml			
+ ve	79 (37.8)	27 (21.1)	0.001
-ve	130 (62.2)	101 (78.9)	
PCT level > 0.5 ng/ml			
+ ve	62 (**29.7**)	16 (12.5)	< 0.001
-ve	147 (70.3)	112 (**87.5**)	
PCT level > 1 ng/ml			
+ ve	49 (**23.4**)	11 (8.6)	0.001
-ve	160 (76.6)	117 (**91.4**)	
PCT level > 2 ng/ml			
+ ve	36 (**17.2**)	9 (7.0)	0.008
-ve	173 (82.8)	119 (**93.0**)	
PCT level > 3 ng/ml			
+ ve	31 (**14.8**)	8 (6.2)	0.01
-ve	178 (85.2)	120 (**93.8**)	

Values are in N(%). *PCT* procalcitonin, *CXR* chest Xray

**Table 5:** Comparison of serum procalcitonin level (at different cut-offs) between non-severe pneumonia (*n* = 211) and severe pneumonia (*n* = 101) (as per WHO criteria)

	Pneumonia (non-severe)	Severe pneumonia	*p*
PCT level > 0.25 ng/ml			
+ ve	60 (28.4)	38 (37.6)	0.1
-ve	151 (71.6)	63 (62.4)	
PCT level > 0.5 ng/mL			
+ ve, *n* = 73	42 (19.9)	31 (30.7)	0.03
-ve, *n* = 239	169 (80.1)	70 (69.3)	
PCT level > 1 ng/ml			
+ ve	30 (14.2)	24 (23.8)	0.03
-ve	181 (85.8)	77 (76.2)	
PCT level > 2 ng/ml			
+ ve	21 (9.9)	19 (18.8)	0.02
-ve	190 (90.1)	82 (81.2)	
PCT level > 3 ng/ml			
+ ve	18 (8.5)	17 (16.8)	0.03
-ve	193 (91.5)	84 (83.2)	

Values are in n (%): *PCT* procalcitonin

The data should be for 312

The sensitivity and specificity of PCT in various combination with the WHO criteria considering any abnormality on chest X-ray as the gold standard for diagnosis of pneumonia, are given in Table 6. The sensitivity and specificity of the existing WHO criteria was 56.5% and 66.2%, respectively (data sent for publication separately). Adding PCT level > 0.5 ng/mL increased the specificity at the cost of sensitivity. If both existing criteria and raised PCT levels were taken as and/or criteria, the sensitivity (57.7%) and specificity (65.9%) did not improve substantially.

**Table 6:** Sensitivity and specificity of combinations of WHO criteria and serum procalcitonin for diagnosis of pneumonia considering any abnormality on chest X-ray as gold standard for pneumonia

**Characteristics**	**Sensitivity**	**Specificity**
Existing WHO criteria, *n* = 1273	331/586 (56.5)	455/687 (66.2)
Existing WHO criteria + Serum procalcitonin levels > 0.5 ng/mL, *n* = 337	55/209 (26.3)	114/128 (89.1)
Existing WHO criteria and/or Serum procalcitonin levels > 0.5 ng/mL; *n* = 1273	338/586 (57.7)	453/687 (65.9)
Existing WHO criteria + ve, Serum procalcitonin levels > 0.5 ng/mL, applied serially, *n* = 297^a^	55/186 (29.6)	97/111 (87.4)

Values are expressed as n(%)

^a^first the children were classified into pneumonia or no pneumonia according to WHO criteria; in children who had pneumonia by WHO criteria, serum procalcitonin levels (PCT) were used to determine presence or absence of pneumonia ( PCT > 0.5 ng/mL: pneumonia, PCT <  = 0.5 ng/mL: no pneumonia)

## Discussion

Although in adults, Procalcitonin as a biomarker for sepsis well established, its role in infections in neonates,children remains less conclusive. CAP is one of the most common infections in children and has been studied extensively because it’s the most frequent cause of hospitalisation and second most common cause of death in children in developing countries [21].

In this study, an attempt was made to find the association between procalcitonin and pneumonia as per both clinical and radiological criteria of WHO. We found positive PCT (> 0.5 ng/mL) was significantly associated with any abnormality on CXR (the gold standard of diagnosis of pneumonia) as well as consolidation on chest X-ray. However, PCT was not significantly associated with the existing WHO criteria for clinical pneumonia. Long et al. reported that chest radiograph has a sensitivity of 46–77%, and biomarkers including white blood cell count, procalcitonin, and C-reactive protein provide little benefit in diagnosis of pneumonia [22]. Korpi et al. evaluated serum PCT in 190 children with radiologically confirmed CAP in a prospective population based study and concluded that there was no useful role for PCT in diagnosis of pneumonia in children in primary health care settings [23]. They concluded that Procalcitonin was poor biomarker to identify radiographic pneumonia (a surrogate for bacterial pneumonia). Using serum procalcitonin may improve specificity but sensitivity remains very low.

In the current study, we used cut off level for PCT as > 0.5 ng/ml. as Some earlier studies have suggested use of a cut-off of > 0.5 ng/mL for identify respiratory tract infections needing antibiotics [19, 20]. However, when we analysed even with lower cut off level(PCT > 0.25 ng/ml),significance was found with any abnormality in CXR and not with clinical pneumonia. Esposito et al. using a 0.25 ng/ml PCT threshold in children found a significant reduction of both antibiotic treatment rate and duration [14]. Conversely, Baer et al., showed no significant reduction in antibiotic treatment rates [24]. A reason for such difference could be that the PCT threshold defined in adult studies (0.25 ng/ml) might be too low for decision making in children with CAP.

In an effort to improve the clinical diagnosis of pneumonia and adding point of care test like PCT levels, we evaluated the sensitivity and specificity of some of the pertinent clinical features, and PCT levels alone or in combination. The sensitivity and specificity of the existing WHO criteria was 56.5% and 66.2%, respectively. Adding PCT level > 0.5 ng/mL increased the specificity at the cost of sensitivity. If both existing criteria and PCT were taken as and/or criteria, the sensitivity and specificity did not change much (57.7%% and 65.9%, respectively). However, when used as part of an algorithm in adults in combination with clinical judgment in patients with LRTIs, procalcitonin has been shown to reduce unnecessary antibiotic use by about 25 to 50% without increasing morbidity or mortality [25–27]. The data regarding children despite being limited, were consistent with those in adults [14]. Dudognon et al. cautioned to promote a rational implementation of PCT especially in children with community acquired pneumonia [28].

We also found that procalcitonin was higher in children with severe pneumonia than in those with non-severe pneumonia. Don et al. and Yadav et al. found that serum PCT was a useful indicator of the severity of CAP in children when comparing the PCT values between those who needed hospitalization and those who did not(*P* < 0.0012), and in comparing the alveolar(*P* < 0.0003) and interstitial pulmonary involvement on chest radiograph [29, 30]. Lee et al. have reported that both PCT and CRP was increased in lobar pneumonia compared to bronchopneumonia; however, only PCT was raised in pneumonia with radiologic finding suggestive of severity [31].

Our findings show that serum PCT values are higher in children with pneumonia with pleural effusion (PE) than in children with pneumonia without pleural effusion. Fonseca et al. reported PE in 10.2% cases. In their study, the PCT level on admission ≥ 1.0 ng/ml was associated with PE [32]. Though the relationship between pleural inflammation and biomarkers has not been well established in children, it has been suggested that pleural inflammation associated with CAP is a continuous process, and inflammatory cytokines are significantly higher according to the stage of disease in the evaluated children [33].

The main strengths of our study were i)prospective collection of samples and ii) multicentric nature involving various regions of the country; however, the study was limited by small sample size in relation to number of pneumonia cases recruited in the study. Additionally, there was lack of use of optimised bacterial diagnostic tests like cultures, antigen detection tests and polymerase chain reaction.

## Conclusion

Positive PCT is significantly associated with radiographic pneumonia but not with pneumonia as per WHO criteria. Adding PCT as a point of care test to existing WHO clinical criteria, didn’t add much value.However, it can act as a surrogate marker for severe pneumonia.

## Data Availability

The datasets used and/or analysed during the current study are available from the corresponding author on reasonable request. All data generated or analysed during this study are included in this published.

## Abbreviations

PCT: Procalcitonin
CAP: Community acquired pneumonia
ATU: Acute respiratory infection treatment unit
ARI: Aacute respiratory tract infection
BPD: Bronchopulmonary Dysplasia
GER: Gastro-oesophageal reflux
HIV: Human immunodefiency virus
CXR: Chest –X-Ray
WHO: World Health Organisation
SpO2: Oxygen saturation
ROC: Receiver operating characteristic curves
PE: Pleural effusion
